# A Time-efficient Multi-Protocol Probe Scheme for Fine-grain IoT Device Identification

**DOI:** 10.3390/s20071863

**Published:** 2020-03-27

**Authors:** Dan Yu, Peiyang Li, Yongle Chen, Yao Ma, Junjie Chen

**Affiliations:** College of Information and Computer, Taiyuan University of Technology, Taiyuan 030024, China; yudan@tyut.edu.cn (D.Y.); lipeiyang0281@link.tyut.edu.cn (P.L.); mayao@tyut.edu.cn (Y.M.); chenjj@tyut.edu.cn (J.C.)

**Keywords:** Internet of Things, device identification, multi-protocol probe, fine-grain identification

## Abstract

Internet of Things (IoT) devices connected to the Internet are exploding, which poses a significant threat for their management and security protection. IoT device identification is a prerequisite for discovering, monitoring, and protecting these devices. Although we can identify the device type easily through grabbing protocol banner information, both brand and model of different types of device are various and diverse. We should therefore utilize multi-protocol probes to improve the fineness of device identification and obtain the corresponding brand and model. However, it is still a challenge to balance between the multi-protocol probe overhead and the identification fineness. To solve this problem, we proposed a time-efficient multi-protocol probe scheme for fine-grain devices identification. We first adopted the concept of reinforcement learning to model the banner-based device identification process into a Markov decision process (MDP). Through the value iteration algorithm, an optimal multi-protocol probe sequence is generated for a type-known IoT device, and then the optimal multi-protocol probes sequence segment is extracted based on the gain threshold of identification accuracy. We took 132,835 webcams as the sample data to experiment. The experimental results showed that our optimal multi-protocol probes sequence segment could reduce the identification time of webcams’ brand and model by 50.76% and achieve the identification accuracy of 90.5% and 92.3% respectively. In addition, we demonstrated that our time-efficient optimal multi-protocol probe scheme could also significantly improve the identification efficiency of other IoT devices, such as routers and printers.

## 1. Introduction

With the development of communications technologies such as LoRa, NB-IoT and 5G [1], more and more Internet of Things (IoT) [2] devices such as routers, webcams, network printers, wearable smart devices, smart home devices and industrial control devices are emerging and exploding in number. According to the statistics of Statista [3], the total amount of Internet of Things (IoT) devices is expected to reach 75.44 billion by 2025. However, IoT devices, connected directly to the physical world, have become a critical attack target for hackers. For example, VPNFilter, appeared in 2018, attacked over 500,000 devices worldwide, including products of Linksys, MikroTik, TPLink, D-link, Huawei, Ubiquiti, UPVEL and ZTE [4]. Therefore, the Internet-connected IoT devices security assessment has become an imperative security measure, while the identification of IoT devices is the prerequisite to execute the security assessment.

For IoT device identification, the predominant method is banner-based identification. The term banner refers to the explicit device attributes contained in the application layer protocol packet of IoT devices, which often include the device type, brand and model [5]. The grabbing of a banner first sends the protocol probe packet of a specific service (i.e., port) to the target device. If the target device opens this specific service, it will return a response packet containing the banner information of the device. Due to the fact a large number of application layer services are typically running, we can grab a wide variety of protocol banners to improve the device identification accuracy. However, we note two deficiencies in banner-based device identification: (1) After sending a protocol probe packet to the target, the target device may not return the corresponding protocol banner; (2) we can extract the device type easily from banners, but most of protocol banners contain incomplete information about device attributes, such as brand and model. Attempts to combine all kinds of protocol banners supported by the device for device identification will raise significant communication and time overhead. Furthermore, the target device possibly take the sharp increase of protocol probes as an intrusion attack [6,7,8]. Therefore, a time-efficient scheduling scheme of protocol probes can well solve the balance problem between protocol probe overhead and device identification fineness, and greatly improve the time efficiency of large-scale IoT device identification.

In this paper, we proposed a time-efficient multi-protocol probe scheme for large-scale fine-grain IoT device identification. Our object is to select the optimal protocol probe sequence for each type of IoT device, which will improve the time efficiency of identifying the brand and model attributes of the IoT device. Through crawling the banners of Internet-connected devices, we first calculate the probability that each protocol banner contains device brand and model. Based on the probability, we model the banner-based device identification process into a Markov decision process. Then we utilize the reinforcement learning method to train the MDP model and generate an optimal protocol probe sequence, where we introduce a new optimal strategy generation method to improve the value iteration algorithm in reinforcement learning. Finally, we design a gain threshold method to extract the optimal protocol probes segment from generated sequence to achieve the balance between the probe overhead and device identification fineness.

In the evaluation, we sent multi-protocol probe packets in order of the optimal protocol probes sequence segment to 132,835 webcams, 109,941 printers and 327,193 routers. The experimental results showed that our time-efficient multi-protocol probes sequence segment could reduce the identification time of webcams’ brand and model by 50.76% and achieve an identification accuracy of 90.5% and 92.3%, respectively. In addition, our time-efficient multi-protocol probe scheme has a better scalability, that is, different types of IoT devices can also use our scheme to obtain a high identification efficiency and accuracy. Our main contributions are summarized as follows:

- We discuss for the first time the balance challenge between protocol probe overhead and identification fineness during banner-based device identification and design a time-efficient multi-protocol probe scheme for fine-grain IoT device identification to solve this challenge.
- We proposed a reinforcement learning method to model the banner-based device identification process into a Markov decision process, and introduce a new optimal strategy generation method to improve the value iteration algorithm so as to generate the optimal multi-protocol probe sequence, and further obtain the optimal multi-protocol probe sequence segment by introducing the gain threshold of identification accuracy.
- We implemented the time-efficient multi-protocol probe prototype system, and generated the optimal multi-protocol probe sequence segment for 132,835 webcams, which could reduce the webcams’ brand and model identification time by 50.76%, and achieved the identification accuracy of 90.5% and 92.3% respectively. Meanwhile, we also verified the scalability of our scheme by evaluating router and printer respectively.

The rest of this paper is structured as follows: Section 2 introduces the work related about IoT device identification. Section 3 describes the design of our time-efficient multi-protocol probe framework. Section 4 collects and analyzes the sample probe data. Section 5 describes in detail our method design. Section 6 is the experimental evaluation. Section 7 summarizes our work.

## 2. Related Work

IoT device identification is the prerequisite to solve a series of IoT device security issues. Traditional fingerprint-based identification technology focuses on the identification of operating system. P0f [9] uses passive probe to detect TCP/IP packets, and puts a network Sniffer [10] at the network boundary to collect data messages and identify the type of operating system. The accuracy and timeliness of identification depend on the network status. SinFP [11] uses a small number of probing and listening packets to identify the operating system by combining active and passive probes, which achieves high identification accuracy. Xprobe [12] uses ICMP packets to extract OS functions. The retransmission time between the target and host can be used as another feature of OS fingerprint identification. Nmap [13] sends a group of 21 probe packets to the target host, and then achieves the identification operating system according to the difference of packet header characteristic fields in the response packet. On the one hand, the identification methods of few operating systems are inapplicable to identify thousands of IoT devices directly, on the other hand, the fingerprint-based method require user to time-consumed manually calibrate packet characteristics from IoT devices.

Banner-based technology is another way to achieve IoT devices identification. Feng et al. [6] analyzed the banner characteristics of 17 industrial control protocols and constructed fingerprints to identify industrial control devices on the Internet. Afterward, they further proposed a rule-based engine (ARE) [14] to annotate industrial control devices by leveraging response banners and product descriptions from websites. Li et al. [15] used natural language processing to extract the response information of Internet-connected webcams, and combined machine learning to construct a classification model to generate device fingerprints automatically. They also designed a device identification system called GUIDE [16], based on the login page features of Internet-connected webcams. It filters out the keyword features of login page through feature extraction method, and then constructs a classifier to identify webcams with a high accuracy. Shaikh et al. [17] proposed a machine learning method to classify and identify malicious IoT devices activities in cyberspace by a dichotomous model. Currently, the device search engines of Shodan [18], ZoomEye [19], and Censys [20] achieved better IoT devices identification and index performance in commercial applications based on banners in the application layer protocol. The Modscan [21] tool for searching SCADA system and the Plcscan [22,23] tool for discovering Siemens PLC devices also use banner information of private protocols for industrial control device identification. Among the methods, most of one protocol banner-based methods result in low identification accuracy, while multiple protocol banner-based methods increase the identification time. Compared with previous works, our object is to greatly improve the time efficiency of identifying device brand and model information without sacrificing the accuracy.

## 3. Time-Efficient Multi-Protocol Probe Scheme

In this section, we introduce our motivation to efficiently and accurately identify the brand and model information of type-known IoT devices. A time-efficient multi-protocol probe framework for fine-grain IoT device identification is designed to generate optimal protocol probe sequences for type-known IoT devices.

### 3.1. Motivation

There is a larger number of application protocols supported by various IoT devices, so we have a wide selection of protocols for device IoT identification. For example, Shodan [18] and Nmap [13] probe packets can cover more than 200 and 1000 protocols, respectively. Among them, the application layer protocols of IoT devices includes three types: common protocols, industry protocols and private protocols. Common protocols represent the predominant Internet protocol, and industry protocols are applied to handle some types of IoT devices, while the private protocols are just used for some brands of IoT devices. These protocols have a large difference in the implementation specifications. In Table 1, we can find that the service protocols and ports run by different types of IoT devices are different, which is determined by the functions they support. For example, webcams don’t have print function, so it does not support service protocols such as IPP and PJL. Meanwhile, some IoT devices can open different ports for one service protocol. For example, an IoT device can open five ports of 80, 81, 8080, 8000 and 8081 to run Http service. Therefore, there will be a lot of alternative probe packets composed of different service protocols and ports for probing IoT devices. In other words, the combinations of probe packets for identifying devices are more than the number of protocols.

**Figure 1:**
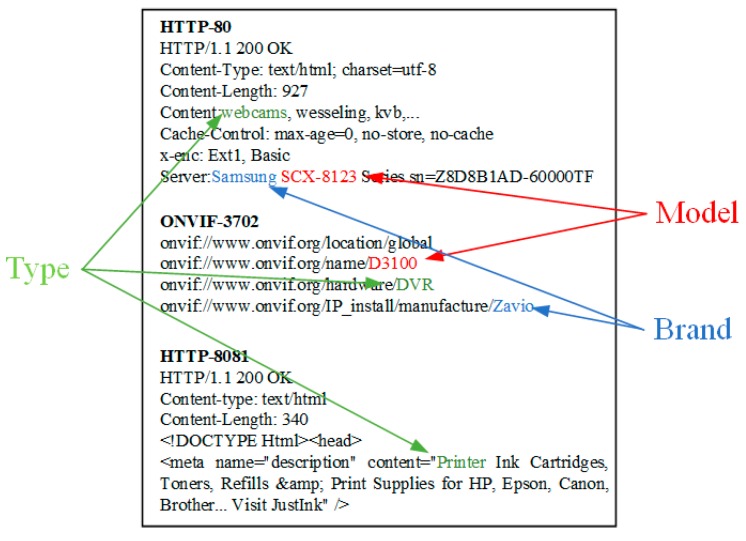
Partial protocol banner information.

Besides, we also find that some protocol probes cannot obtain the corresponding banner from IoT devices. Aside from probe or response packet loss due to network jitter, one possible reason is that the target device does not open the corresponding service and port. However, even if the banner is obtained, the device attribute information in the banner may be incomplete. Although most of banners contain device type, it is possible that some banners do not contain brand or model attribute as shown in Figure 1.

We also have verified this fact by real data in Section 6. Therefore, although there are many alternative protocol probes, the simple method of increasing the number of protocol probes to improve the identification accuracy will lead to excessive time cost and communication overhead. On the contrary, few protocol probes will also affect the identification accuracy. In order to solve this problem, we intend to design and implement a scalable time-efficient multi-protocol probe scheme by exploring the banner-based device identification process. We utilize this scheme to generate the optimal protocol probe sequence for type-known IoT devices, which reduce the time cost to identify the device brand and model significantly and improve the identification efficiency of Internet-wide IoT devices.

### 3.2. Time-efficient Multi-Protocol Probe Framework

**Figure 2:**
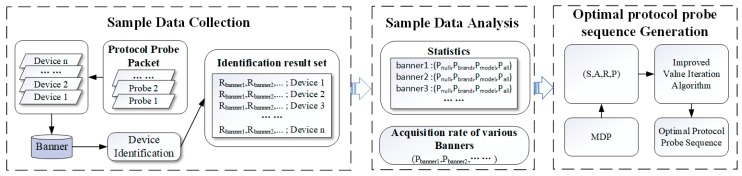
Time-efficient multi-protocol probe framework.

In order to improve the identification efficiency of the brand and model of type-known IoT devices, we designed a time-efficient multi-protocol probe scheme for fine-grain devices identification as shown in Figure 2. The framework is divided into three modules: sample data collection, sample data analysis and optimal protocol probe sequence generation.

The sample data collection module includes constructing the probe packets combined by protocols and ports, grabbing the corresponding banners and performing banner-based devices identification based our attribute fingerprint database. We record the identification result of each protocol banner for further analysis.

The sample data analysis module calculate the banner acquisition rate $\left(P_{Banner1},P_{Banner2},\ldots \right)$ of each protocol banner for a type-known device, and then identify device brand and model information to count four identification probabilities $\left(P_{null},P_{brand},P_{model},P_{all}\right)$, which refer to the protocol banner information with no attributes (null), only device brand (brand-only), only device model (model-only), and both device brand and model (all), respectively.

The optimal protocol probe sequence generation module adopts reinforcement learning method to solve the problem of balance between multi-protocol probe overhead and device identification fineness. We modeled the banner-based identification process as a Markov Decision Process (MDP) to find the optimal protocol probe strategy. The strategy maps the state to the action of sending protocol probe packets, so that the cumulative reward is maximized. Secondly, according to the data analysis results obtained in the sample data analysis module, the state transition probability set in the MDP is calculated. Finally, we designed a value iteration algorithm. The state set, action set, state transition probability set, and immediate reward value in our MDP model are used as the input of our algorithm to generate optimal protocol probe sequences for type-known IoT devices.

## 4. Sample Data Collection and Analysis

In this section, we first describe the banner-based device identification process. Then, we take the webcam device as an example to introduce how to build a sample data set for analyzing the identification process. Finally, the sample data is statistically analyzed for modeling the identification process of type-known IoT devices.

### 4.1. Banner-Based Device Identification Process

**Figure 3:**
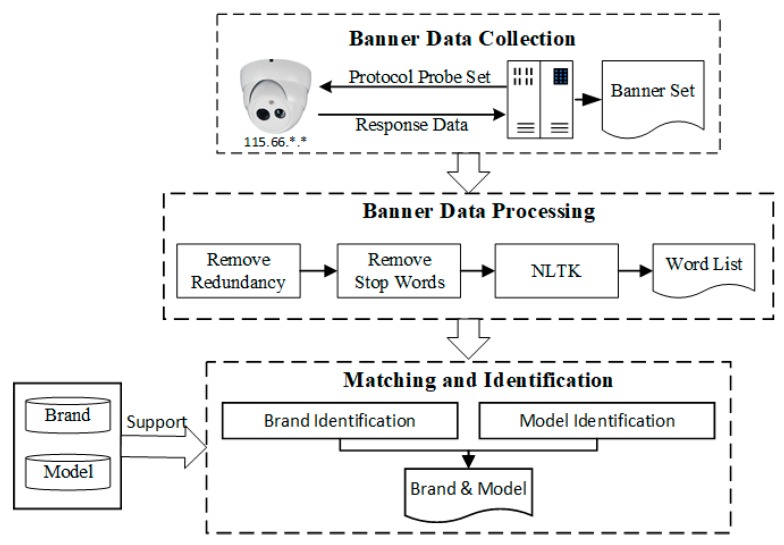
Banner-based webcams identification process.

The process of banner-based webcams identification is shown in Figure 3, which is divided into three phases: banners data collection, banner data processing and matching identification. In the banner data collection phase, the probe host actively sends protocol probe packets to the IoT device to obtain different kinds of protocol response banner information. Banner data processing utilize Natural Language Toolkit (NLTK) to remove non key factors such as stop words and special symbols in the protocol banners and segment the banners information to generate the segmentation list. The matching identification process uses the device fingerprint database with brand and model attribute to filter the segmentation list, and finally obtains the brand and model information of the device by keywords matching.

### 4.2. Sample Data Collection

#### 4.2.1. The Construction of an IoT Device Fingerprint Database

**Figure 4:**
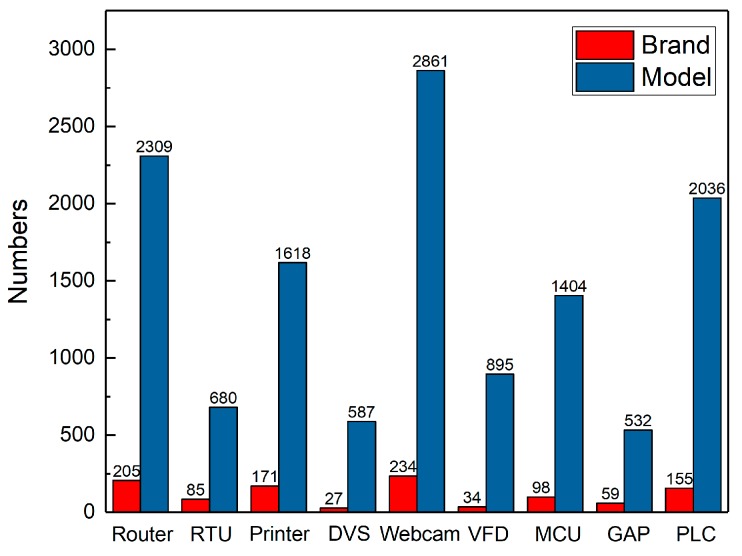
Device brand and model statistics.

The purpose of building the device fingerprint database is to filter and match the device attribute information in the device protocol banner information, so as to judge whether the protocol banner contains the brand and model information of target device. Each item in fingerprint database include three attributes of the device type, brand and model. Besides, the automatic construction of the IoT device fingerprint database can reduce the manual participation in the collection of brands and models so as to improve the collection efficiency. In general, for reducing labor consumption, the attribute information of IoT device can be found on the official websites of manufacturers. However, searching on each manufacturer’s website is also time-consuming. We find the website of Internet retailers (e.g. Amazon, Taobao and eBay) contains well structural information of IoT devices attribute. Therefore, according to the structural features of brand and model displayed on the websites, we achieve the automatic collection of IoT device attributes based on Python Scrapy [24] architecture. The automatic crawling of device brand and model has good scalability so that our IoT device fingerprint database can be updated periodically. Finally, we collect a lot of brand and model information about webcam, network printer, router and other devices, as shown in Figure 4.

#### 4.2.2. The Results of IoT Device Identification

In Table 1, the open ports and running services of different types of IoT devices are different, especially in the industry protocol and private protocol supported by the devices. In this section, take the webcam as an example, according to the services protocols and ports in the webcam device shown in Table 1, ten kinds of protocol probe packets are constructed by combining of service protocols and ports to probe the webcam device. In order to fully explore the contribution of each protocol to the identification of brand and model information of webcams, we send all the ten protocol probe packets to webcams in order to obtain the corresponding protocol banner information. After executing the banner-based device identification process as shown in Figure 3, the identification results for each combination of protocol and port are obtained. We utilize a two tuples to formalize two attribute values of brand and model, where (0,0) means that the corresponding protocol banner information does not contain any attribute of the device, (1,0) means only containing the brand information, (0,1) indicates that only the model information is included, and (1,1) indicates that both brand and model information are in the banner. If a target device does not return a response protocol banner at all, we mark the corresponding identification result as (0,0). We save all the identification results in the sample data set.

### 4.3. Sample Data Analysis

Suppose sending ten protocol probe packets $\left(l_{1},l_{2},\ldots ,l_{10}\right)$ to *N* webcams to obtain the corresponding protocol banner information, we will get the number of devices that can return protocol banner is $\left(n_{1},n_{2},\ldots ,n_{10}\right)$ respectively. So the banner acquisition rate $P_{Banner\_i}$ of the *i*th protocol banner for webcam is calculated by Equation (1):

(1) $$P_{Banner\_i}=\frac{n_{i}}{N},\left(i∊\left[1,10\right]\right)$$

Through statistical analysis on the number of devices attributes contained in each protocol banner, we can calculate four attribute proportion rate $\left(P_{null},P_{brand},P_{model},P_{all}\right)$, which represent the probability that a protocol banner with no attributes (null), only device brand (brand), only device model (model), and both device brand and model (all).

**Figure 5:**
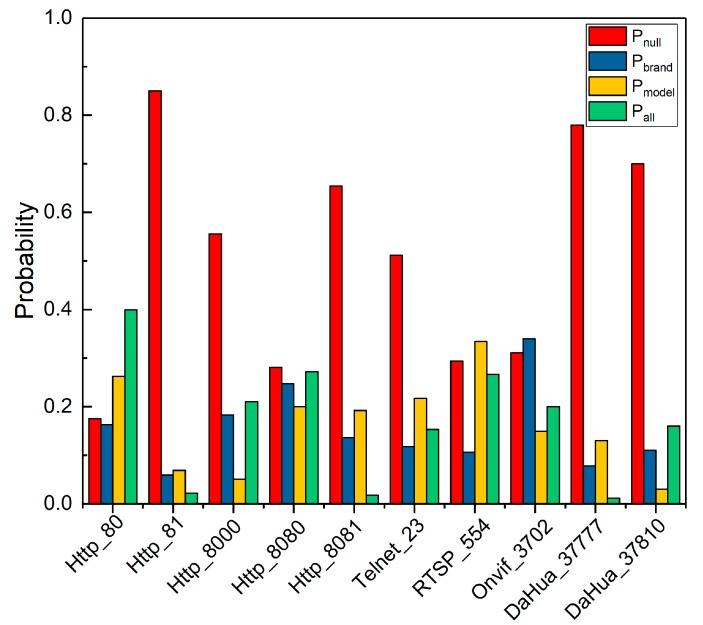
Attributes proportion rate of ten Webcam protocol banners.

Figure 5 shows attribute proportion rate of ten protocol probes of webcam. Meanwhile, we also found the following identification features:

1) Among the ten protocol banners to identify webcam, the contained attribute information is complementary. E.g. if a protocol banners does not contain one attribute of the device, the other nine protocol banners have a higher probability of containing this attribute.
2) The probability of identifying device brand and model by only one protocol banner is low. In the process of identifying IoT devices, multiple protocols probe packets are required to jointly probe devices, so as to obtain protocol banners with richer device attributes.
3) Without considering the banner acquisition rate, the $\left(P_{null},P_{brand},P_{model},P_{all}\right)$ corresponding to each protocol banner is different. The protocol banner of Http_80 contains device attribute with the probability of more than 80%, while the protocol banner of DaHua_37777 and Http_81 contains the device attribute with the probability of less than 30%.

In short, it is practical and necessary to improve the efficiency of device identification by scheduling multi-protocol probes.

## 5. Optimal Multi-Protocol Probe Sequence Generation

We further count the combination identification probability of different protocol banners in Figure 5. We can find different combinations have different protocol complementarity for identifying device attributes. For example, using two protocol banners of Http_80 and RTSP_554, we can obtain the device model probability of 66.2% and 58.7% respectively, and combine the two protocols to obtain device model probability of 71%. Although the Onvif_3702 protocol banner can only obtain the device model probability of 34.2%, which is far less than the device model probability of RTSP_554 protocol banner, it can achieve 83% of the devices model identification probability after combining with Http_80 protocol banner. In other words, the complementarity between Http_80 and Onvif_3702 is much higher than that between Http_80 and RTSP_554. The reason for this difference is that the device model identification results from the combination of Http_80 and RTSP_554 protocol banners have many duplicate devices. Therefore, by optimizing the combination of multiple protocols and forming the optimal multi-protocol probes scheduling sequence, the communication overhead and identification time can be significantly reduced.

In this section, we mainly discuss how to generate optimal multi-protocol probe sequence for type-known IoT devices. We first analyze the whole banner-based device identification process and transform the generation process of the multi-protocol probe sequence of IoT devices into Markov decision process based on reinforcement learning concept. Finally, we optimized the strategy generation method in the value iteration algorithm and used it to generate an optimal multi-protocol probe sequence for type-known IoT device.

### 5.1. Scheduling Model of Multi-Protocol Probe Sequence

It is a scheduling problem to construct the optimal multi-protocol probe sequence of IoT devices. In the process of identifying device brand and model information based on banners, according to the findings in Section 4.3, we need to send *n* different types of protocol probe packets one by one to obtain the corresponding protocol banners. The optimal protocol probe sequence of device is to get the protocol banners which contain more attribute information so as to identify the brand and model of device faster and reduce the time cost and communication overhead. Each protocol banner has different benefits for device identification, and the different orders of sending protocol probe packets will lead to dynamic changes in identification benefits.

**Figure 6:**
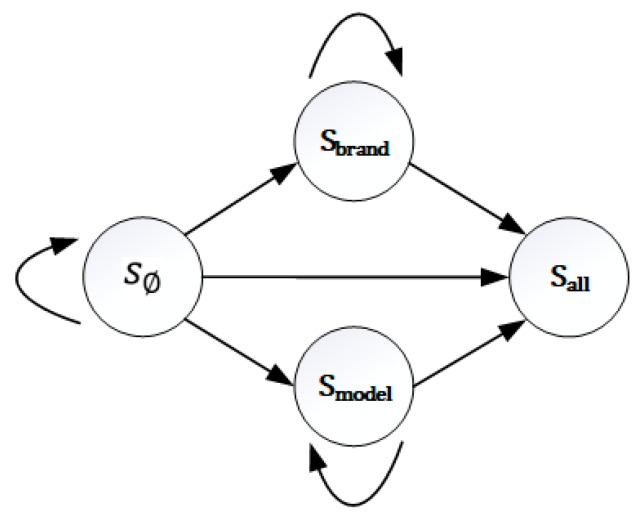
Identification state transition diagram.

We use reinforcement learning method to construct the model of device identification process. Reinforcement learning is a kind of decision-based learning method developed from adaptive control theory. In decision process, agent is used to count the learning results in the current interactive context, and these results are taken as the feedback to keep continuous learning in order to obtain the optimal decision process. The goal of reinforcement learning is to give a Markov decision process (MDP) and find the optimal strategy. In this paper, the corresponding action of MDP model is to send protocol probe packet to the device. Firstly, based on the Markov decision process, the formal analysis of device identification process is carried out. In the Markov decision process, the agent can perceive the different state set *S,* and has its executable action set *A*, which refers to all protocol probe packets sets. At each discrete time *t*, the agent perceives the current state $s_{t}$, chooses to perform the current action $a_{t}$, and obtains the return $r_{t}=\tau \left(s_{t},a_{t}\right)$, and then generates a successor state $s_{t+1}=\delta \left(s_{t},a_{t}\right)$. The generation of the successor state is only related to the current state. In this paper, the symbol *S* represents the set of identification states corresponding to all times. There are only 4 types of identification states$\;\left\{s_{\emptyset },s_{brand},s_{model,}s_{all}\right\}$, where $s_{\emptyset }$ indicates that the device has no attribute information identified, $s_{brand}$ indicates that only the device brand is identified, and $s_{model,}$ indicates that the device model is identified, $s_{all}$ means that the brand and model of the device have been identified and the identification process is stopped. The state transition process is shown in Figure 6.

Symbol *a* represents the currently sent protocol probe packet. Symbol *r* means the immediate reward value provided by the change of the device identification status after sending the protocol probe packet. If the device identification status does not change, *r* = −1. If only one brand or model attribute information of the device is added, *r* = 1. If both brand and model attributes of the device are identified, *r* = 2. δ represents the state transition function. The task of the agent is to learn a strategy: π:S→A, which selects the next action $a_{t}$ based on the currently observed state $s_{t}$, that is, $\pi \left(s_{t}\right)=a_{t}$. Meanwhile, for any state $s,s^{\prime }$ and action a, there is a probability $p_{s\to s^{\prime }}^{a}$ when the action a is transferred to the state $s^{\prime }$ under the state s, which is calculated by the acquisition rate of each protocol banner and its corresponding device attribute proportion rate. When device identification is based on each protocol banner, the calculation formula of its corresponding state transition probability matrix P is shown in Table 2. In the table, to simplify the symbol, $\;p_{B}$ means protocol banner acquisition rate, $p_{n}$ means the probability that there is no device attribute in protocol banner, $p_{b}$ and $p_{m}$ mean the probability that protocol banner contains only device brand or model, and $p_{a}$ means the probability that both device brand and model are included in the protocol banner.

Therefore, the state transition probability matrix $P=\left\{P_{1},P_{2},\ldots ,P_{i},\ldots P_{n}\right\}$ is formed by device identification based on *n* protocol banners.

### 5.2. Value Iteration Algorithm

Due to both the transition probability *P* of the identification states and the immediate reward value *R* of the action are known in our model, it is a model-based reinforcement learning. Therefore, for any strategy π, the expected cumulative reward brought by the strategy can be estimated. Let the value function $V^{\pi }\left(s\right)$ represents starting from state s and using the cumulative reward brought by strategy π. Because MDP has Markov properties, that is, the next state of the system is determined only by the current state, so there is a recursive expression as shown in Equation (2):

(2) $$V_{\gamma }^{\pi }\left(s\right)=\underset{a\epsilon A}{\sum }\pi \left(s,a\right)\underset{s\prime ∊S}{\sum }P_{s\to s\prime }^{a}(R_{s\to s^{\prime }}^{a}+\gamma V_{\gamma }^{\pi }\left(s\prime \right)$$

where 0 ≤ γ < 1 is a constant, called the discount factor, which determines the relative proportion of delayed reward and immediate reward. When the value of γ is 0, it means that only the immediate reward is considered. The current optimal multi-protocol probe strategy π can be selected by calculating the maximum discounted cumulative reward value, i.e. maximizing $V^{\pi }\left(s\right)$. The optimal strategy $\pi^{*}$ can be expressed by Equation (3):

(3) $$\pi^{*}=argmax_{\pi }V^{\pi }\left(s\right),\left(\forall s\right)$$

The value function corresponding to the optimal strategy is $V^{\pi^{*}}\left(s\right)$, abbreviated as $V^{*}\left(s\right)$, which means the maximum cumulative conversion reward obtained starting from the state s, that is, the cumulative conversion reward is obtained by the optimal multi-protocol probe strategy starting from the state s.

Starting from state s, after taking an action a and executing the optimal strategy $\pi^{*}$, we use the state-action function $Q^{\pi }\left(s,a\right)$ to express the cumulative reward. Its recursive form is expressed in Equation (4). For the state *s*, executing different actions will obtain different *Q* values, then the optimal action *a* starting from state *s* can be calculated by Equation (5):

(4) $$Q_{\gamma }^{\pi }\left(s,a\right)=P_{s\to s^{\prime }}^{a}\left(R_{s\to s^{\prime }}^{a}+\gamma V_{\gamma }^{\pi^{*}}\left(s\prime \right)\right)$$

(5) $$a^{*}=argmax_{a∊A}Q^{\pi }\left(s,a\right)$$

We can find that it is actually a dynamic programming algorithm to use the recursive equations of Equations (2) and (4) to calculate the value functions $V_{\gamma }^{\pi }$ and $Q_{\gamma }^{\pi }$ respectively. For $V_{\gamma }^{\pi }$, since $\gamma^{t}$ tends to 0 when *t* larger, it is finite to continue the recursion until the starting point. In addition, in order to obtain $V^{*}\left(s\right)$, it may be iterated many times, so we set a threshold θ to limit it. If the value function changes less than θ after one iteration, the iteration is stopped:

(6) $$max_{s∊S}\left|V\left(s\right)-V^{\prime }\left(s\right)\right|<\theta$$

In the process of generating the optimal protocol probe sequence, the $V^{*}\left(s\right)$ of each state is calculated iteratively to maximize the cumulative reward value of the optimal value function. In this way, the sum of actions in Equation (2) can be transformed to the maximum in Equation (7):

(7) $$V_{\gamma }^{*}\left(s\right)=max_{a\epsilon A}\underset{s\prime ∊S}{\sum }P_{s\to s^{\prime }}^{a}\left(R_{s\to s^{\prime }}^{a}+\gamma V_{\gamma }^{\pi }\left(s\prime \right)\right)$$

The traditional value iteration algorithm only focuses on the optimal actions in each state. If it is directly applied to the process of generating the optimal protocol probe sequence, it is possible to get the same optimal action for each device identification state and result in only one protocol in the final protocol probe sequence. The reason is that if different identification states do not achieve the termination state, it is necessary to select an optimal action from the action set to continue probe. Due to the corresponding action set of each state is the same, it is easy to cause the selected optimal action to be the same or the action has been executed before the selection, so as to get a dead cycle.

In order to avoid this situation, we optimized the strategy generation method in the value iteration algorithm. After calculating the *Q* (*s, a*) of each action corresponding to each state, the actions in the action set are sorted according to the *Q* value from large to small, and the optimal protocol probe sequence *seq* corresponding to each identification state is obtained. Finally, according to the sequence of identification states $\left\{s_{\emptyset },s_{brand},s_{model,}s_{all}\right\}$ and *seq*, the corresponding actions in the sequence *seq* of each state are selected in turn and repeatedly, and the optimal multi-protocol probe sequence of a type-known IoT device is obtained. According to the related works [25,26], it can be proved that the algorithm has convergence, that is, the optimal protocol probe sequence obtained is unique. Due to space limitation, the proof process is not given here. The specific algorithm description is shown in Algorithm 1.
**Algorithm 1** Value iteration algorithm**Input:** E(S, A, P, R); S’; γ; θ
//E(S, A, P, R): MDP quad//S’: Special Identifying status set//γ: Discount factor//θ: Convergence threshold**Output:** Optimal_Sequence1  ∀s∊S:V(s)=0;2  **for** t=1,2,… **do**3  ∀s∊S:
$V\left(s\right)=max_{a∊A}\underset{s\prime ∊S}{\sum }P_{s\to s^{\prime }}^{a}\left(R_{s\to s^{\prime }}^{a}+\gamma V\left(s\prime \right)\right)$;4  **if** max s∊S |V(s)-V’(s)|<θ **then**5  break;6  **else**7  V=V’;8  **end if**9  **end for**10  **for** i∊S’ **do**11 **for** j∊A **do**12  $Q\left(i,j\right)=P_{s\to s^{\prime }}^{a}\left(R_{s\to s^{\prime }}^{a}+\gamma V\left(s\prime \right)\right)$;13  **end for**14  //Sort a by Q15  $seq_{i}=sort\left(Q\left(i\right)\right);$
16  **end for**17  Sequence=combine$\left(seq_{\emptyset },seq_{brand},seq_{model}\right)$;18  **return** Sequence;

Besides, in order to achieve a balance between identification time and identification fineness, we set a accuracy gains threshold to truncate the sequence. In practice, we believe that if the accuracy gains on brand or model by increasing a protocol probe is less than the threshold of 1%, the subsequent protocol probe packets will be discarded. Finally, we will generate a segment of optimal multi-protocol probe sequence. For other IoT device types, such as router and printer, we can also generate the optimal protocol probe sequence segment.

## 6. Results

In this section, we first describe the prototype system implementation and the experiment settings. Afterward, we show the experimental results of evaluating our time-efficient multi-protocol probe scheme, where the evaluation metric includes identification accuracy, identification time efficiency and scheme scalability. We evaluate the identification accuracy and time by using webcams and verify the scalability of our scheme by using printers and routers.

### 6.1. Prototype System Implementation

We implemented and deployed our experimental prototype system on a commercial desktop computer (64-bit Windows 10, i5-8500, 3.0Hz, 16GB memory) [27,28]. In order to reduce identification time for whole Ipv4 cyberspace, we crawl a part of Internet-connected IoT devices in Censys. By calling Censys API interface, we filter three types of IoT devices, webcams, printers and routers. Meanwhile, we use Zmap [29] to check if these device hosts are all surviving, and obtain 132,835 webcams, 109,941 printers, 327,193 routers. However, the banners in Censys do not contain all the protocol probe combinations of IoT devices. We use Zgrab2 [30], a banner grabber, to grab a variety of protocol banners for the device, but the open source Zgrab2 supports only a few types of protocols, and lacks especially industrial and private protocols such as ONVIF, RTSP, DaHua. Therefore, by resolving the protocol fields, we implement a Zgrab2+ based on the Zgrab2 to support all protocols listed in Table 1. By using Zgrab2+, we can crawl all the protocol banners of 132,835 webcams, 109,941 printers and 327,193 routers as sample dataset. Meanwhile, we also implement a banner-based devices identification system to handle banner sample dataset. Taking webcams for instance, we first send ten kinds of protocol probe packets to webcams by Zgrab2+ in order to obtain corresponding banners. Second, we remove the redundant parts of the protocol banners, such as “\r \n” in the Telnet protocol banners, and remove special symbols, punctuation marks, and unprintable characters, and then further to filter the characters obfuscated device model, such as dates. Furthermore, we use natural language processing method for the remaining protocol banners. We delete the English stop words in the NLTK library and use the tokenize word segmentation tool in NLTK library to obtain the banner vocabulary list. Finally, the device brand and model information contained in the protocol banners was obtained by matching with the attribute fingerprint database generated in Section 4.2. 

In this paper, we average divide sample dataset of IoT devices into ten parts. The nine parts are used to train and generate the optimal time-efficient multi-protocol probe sequence, and another one part is used to test the performance of our scheme. We need to manually calibrate the brand and model in test dataset as our identification benchmark. But the scale of manual calibration for a tenth of sample is still larger. Therefore, we first compare our identification results of brand and model with Censys, then we just filter the inconsistent results to execute manual calibration process, which will significantly reduce our workload.

In addition, we first make a test to explain the reason why we focus on device brand and model to generate multi-optimal protocol probe sequence is that most of the protocol banners can contain device type but contain incomplete device brand and model information. We use an experiment to verify this fact. In the experiment, we selected the two predominant protocols of webcams, Http and Onvif, and utilized Http_80 and Onvif_3702 to grab the banners of 10,000 webcams in test dataset. Afterword, we extract and analyze the device type, brand and model proportion in each protocol banner. In Table 3, we find more than 90% protocol banners of webcams contains the device type attribute, while almost a half of brands and models in both protocol banners can not be identified. Therefore, fine-grain identification of device brand and model is our challenge need to be solved.

### 6.2. Performance Evaluation

#### 6.2.1. Identification Accuracy

**Figure 7:**
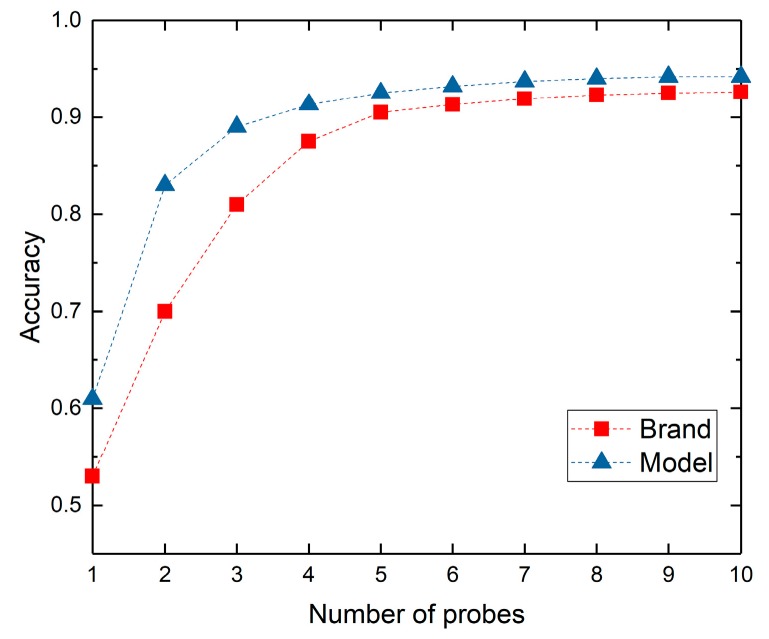
The identification accuracy variety with the increasing of protocol probes in order.

For 132,835 sample webcams, we select 119,552 webcams to generate the optimal multi-protocol probe sequence segment including the top five probes of the optimal multi-protocol probe sequence, namely Http_80, Onvif_3702, Http_8080, RTSP_554 and Telnet_23. If we increase the type of protocol probe packet, the gains of the brand and model identification accuracy will be less than the gain threshold of 1%. Therefore, we do not use the last five protocol probe packets in the optimal multi-protocol probe sequence in order to reduce the identification time. In Figure 7, the *x*-axis represents the number of protocol probes that is sequentially increasing in order of the optimal multi-protocol probe sequence <Http_80, Onvif_3702, Http_8080, RTSP_554, Telnet_23, DaHua_37810, Http_8000, DaHua_37777, Http_8081, Http_81>. The *y*-axis indicates the identification accuracy. We found that the impact of the increasing protocol types on the identification accuracy is becoming smaller and smaller. There are two reasons. On the one hand, the brand and model information of most devices have been included in the top several protocol banners in the optimal protocol probe sequence. On the other hand, private protocols such as DaHua only support their own brand products, they have little impact on the identification of other IoT devices. Therefore, when we identify the large-scale type-known IoT devices, if we pursue time efficiency, we can ignore some protocol probes with gain less than 1% in the tail of optimal sequence. If we want to achieve high identification accuracy, we can increase the protocol probes in order of the optimal protocol probe sequence.

Table 4 provides a comparison of our approach with others. It shows that our brand and model identification accuracy can achieve 90.5% and 92.3% respectively only by top five protocol probes in the optimal multi-protocol probe sequence, superior to the firmware identification [15] and type identification [31,32]. Although our performance is less than the type identification of 95%, our identification fineness is far higher than type identification method. Meanwhile, we only select the top five protocol probes in the optimal multi-protocol probe sequence, which will reduce the probe time significantly.

#### 6.2.2. Time Efficiency

In order to explore how our optimal multi-protocol probe sequence segment can improve the efficiency of device identification, we calculated the time cost to identify the brand and model of 13,283 webcams with the increasing of protocol probes in order of the optimal multi-protocol probe sequence. We compared our scheduling scheme with the method [33] of device identification based on the popularity of protocol.

**Figure 8:**
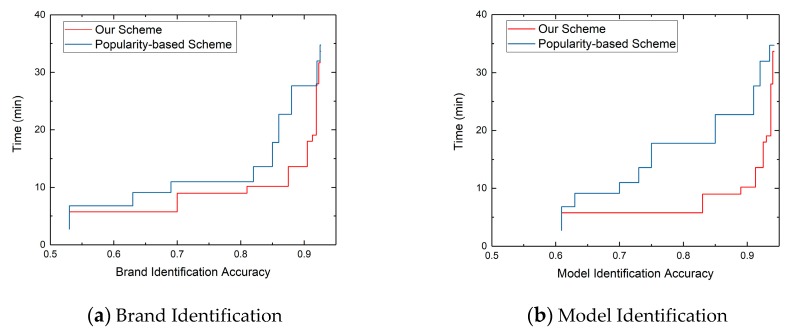
The relationship between identification accuracy and identification time.

In Figure 8, we show the relationship between the identification accuracy and identification time of our scheme and popularity-based scheme. Figure 8a,b show the brand and model identification respectively. We can find when the identification accuracy of brand and model is 90%, the time required for device brand and model identification based on our scheduling method is 13.62 minutes, while the time required for device brand and model identification based on protocol popularity-based method is 27.66 minutes. Our scheme can save 50.76% identification time in identifying device brand and model.

**Figure 9:**
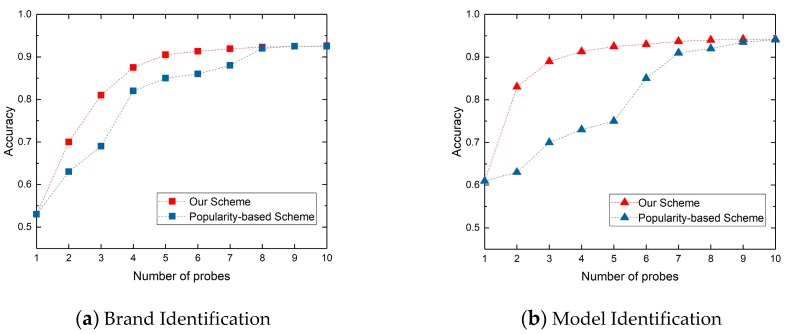
The comparison of identification accuracy between our schemes with others.

We further show the reasons for the reduction of identification time in Figure 9, which shows the variety of the identification accuracy obtained by different protocol probe sequences. The red line is obtained from our optimal multi-protocol probe sequence < Http_80, Onvif_3702, Http_8080, RTSP_554, Telnet_23, DaHua_37810, Http_8000, DaHua_37777, Http_8081, Http_81 >, and the blue line is obtained from the popularity -based protocol probe sequence < Http_80, Http_81, Http_8000, Http_8080, Http_8081, RTSP_554, Onvif_3702, Telnet_23, DaHua_37777, DaHua_37810 >. The *x*-axis represents the number of protocol probes, and the *y*-axis represents the identification accuracy. We can find that our scheduling scheme is better than popularity-based scheme in banner-based device identification accuracy, especially for model identification. We can achieve the 90% accuracy of brand and model with five protocol probes, while popularity-based scheme needs eight protocol probes to achieve 90% accuracy.

#### 6.2.3. Scalability

Our scheme can not only generate the optimal multi-protocol probe sequence for webcam devices, but also be successfully applied to other types of IoT devices. In order to verify the scalability of our scheme, for 109,941 printers and 327,193 routers, we use our time-efficient multi-protocol probe scheme to generate the optimal protocol probe sequence for routers and printers respectively. According to the gain threshold of identification accuracy, the protocol probe sequence segment with top 4 is selected for two types of IoT devices. We use the protocol probe sequence segment of each type of IoT device to calculate the identification accuracy and identification time before and after scheduling our multi-protocol probe scheme. We define time reduction ratio to describe the changing of identification time. As shown in Table 5, the identification time reduced by 35% and 52% for routers and printers respectively. Meanwhile, the identification accuracy still achieves more than 90%. the larger time reduction ratio indicate that our optimal multi-protocol probe scheme is more time-effective for improving the router and printer identification. Therefore, with the better scalability, our scheme can also generate optimal protocol probe sequence for other types of IoT devices.

## 7. Conclusions

In this paper, we designed a time-efficient multi-protocol probe scheme to identify the type-known IoT devices. In order to improve the identification efficiency of large-scale IoT devices, we explored the banner-based device identification process through statistical calculation of sample data and adopted the concept of reinforcement learning to model the banner-based device identification process into a Markov decision process (MDP). In addition, by optimizing the value iteration algorithm, we generated an optimal multi-protocol probe sequence, and then we further extracted a segment of protocol probe sequence to reduce identification time based on the gain threshold of identification accuracy. The experimental results showed that our optimal time-efficient multi-protocol probes sequence segment could reduce the webcams’ brand and model identification time by 50.76% and achieve the identification accuracy of 90.5% and 92.3%, respectively. In addition, we demonstrated that our scheme could also improve the identification efficiency of routers and printers.

## Figures and Tables

**Table 1:** Predominant IoT devices and their service protocols and ports.

Device Type	Protocol Type	Ports
Webcam	Http	80,81,8000,8080,8081
	Telnet	23
	Rtsp	554
	Onvif	3702
	DaHua	37777,37810
Router	FTP	21
	SSH	22
	Telnet	23
	DNS	53
	Http	80,8080,8081
	UPnP	1900
Printer	Http	80,8000,8080,8081
	IPP	631,4567
	SNMP	161
	SSH	22
	Telnet	23
	PJL	1900

**Table 2:** State transition probability matrix.

	Next			
Start	$s_{\emptyset }$	$s_{brand}$	$s_{model}$	$s_{all}$
**$s_{\emptyset }$**	$1-P_{B}*\left(1-P_{n}\right)$	$P_{B}*P_{b}$	$P_{B}*P_{m}$	$P_{B}*P_{a}$
**$s_{brand}$**	0	$1-P_{B}*\left(1-P_{n}-P_{b}\right)$	0	
**$s_{model}$**	0	0	$1-P_{B}*\left(1-P_{n}-P_{m}\right)$	$P_{B}*(P_{b}+P_{a})$
**$s_{all}$**	0	0	0	1

**Table 3:** The device attributes identification of Onvif and Http protocol.

Protocol Probe	Probability of Type	Probability of Brand	Probability of Model
Onvif_3702	91.00%	54.00%	34.9%
Http_80	92.97%	56.25%	65.16%

**Table 4:** Comparison of identification accuracy with others

Author	Identification Objects	Method	Accuracy
Li et al. [15]	Firmware	Web Crawler & NLP	90%
Hamad et al. [31]	Device Type	ML	90.3%
Aksoy et al. [32]	Device Type	ML	95%
Our Scheme	Device Brand & Model	Banner Grab	90.5%, 92.3%

**Table 5:** Other types of IoT devices identification by our scheme.

Device Type	Number of Probes	Identification Time after Scheduling(min)	Identification Time before Scheduling(min)	Time Reduction Ratio	Identification Accuracy
Router	4	13.73	21.2	0.35	91.5%
Printer	4	12.52	25.9	0.52	90.02%
